# The role of action inhibition for behavioral control in joint action

**DOI:** 10.3758/s13423-022-02162-5

**Published:** 2022-08-15

**Authors:** Martin E. Maier, Roman Liepelt, Marco Steinhauser

**Affiliations:** 1 Department of Psychology, Catholic University of Eichstätt–Ingolstadt, Ostenstraße 25, D-85072 Eichstätt, Germany; 2 Department of General Psychology: Judgment, Decision Making, Action, Faculty of Psychology, University of Hagen (FernUniversität in Hagen), Universitätsstraße 27, D-58097 Hagen, Germany

**Keywords:** Social cognition, Action observation, Joint Simon task, Conflict adaptation, Response inhibition, Task shaping

## Abstract

When two individuals share a task with a common goal, coordinating one’s own and the other’s actions is pivotal. Inhibition of one’s own actions when it is the other’s turn to act is assumed to play a crucial role in this process. For instance, in the joint Simon task, two individuals share a two-choice task such that one of them responds to one stimulus type and ignores the stimulus type to which the other responds. Because stimuli can either appear on one’s own or on the other’s side, stimulus location can conflict with stimulus identity, thus slowing response time. It has previously been shown that such conflict leads to a reduction of the detrimental effects of conflict on immediately upcoming trials both following own responses and even more so following the other’s responses. This amplified trial-to-trial adjustment following the other’s responses has been assumed to reflect the inhibition of own responses on the other’s trials. The present study tested this hypothesis by comparing sequential trial-to-trial adjustments following correct responses and commission errors on which the inhibition of own responses has failed. As expected, adjustments were stronger following the other’s correct responses than following own correct responses. Crucially, such amplification of sequential adjustment was not observed following own commission errors on the other’s trials. This shows that amplification of sequential adjustments following the other’s trials depend on successful inhibition of own responses on these trials and points to a crucial role of response inhibition for behavioral control in joint action.

## Introduction

Assembling furniture with another person, sharing child care between two working spouses, or writing a research article with a group of scientists are typical examples of several people sharing a task with a common goal. This requires one to integrate and coordinate one’s own actions with the other’s actions. This process can be studied in the so-called joint Simon task (see Dolk et al., 2014, for a review). Two participants seated side by side respond to one specific nonspatial stimulus feature each (e.g., one participant responds to blue stimuli, the other participant responds to green stimuli). Stimulus position is compatible or incompatible with each participant’s side, and although stimulus position is irrelevant for the task, spatial incompatibility slows response time (RT). This joint Simon effect (JSE) is often contrasted with a condition where the task is performed alone as a go/no-go task, where stimulus side does not affect RT (e.g., Dolk et al., 2011; Dolk et al., 2013; Sebanz et al., 2003).

To explain the JSE, one hypothesis was that the other’s actions are treated functionally in the same way as one’s own actions (Sebanz et al., 2003). This action co-representation account assumes that in joint task performance, each actor fully integrates the co-actor’s task into their own task set (e.g., Knoblich & Sebanz, 2006; Sebanz et al., 2003). Therefore, spatial incompatibility of response side and stimulus location evokes response conflict just as if one person was performing the task alone as a two-choice Simon task (Sebanz & Knoblich, 2009; Sebanz et al., 2003; Sebanz et al., 2005). Another account of the JSE originated from ideomotor theories of action control, which hold that action selection operates through activation of codes representing the perceivable consequences of actions (Hommel et al., 2001). This referential coding account (Dolk et al., 2014; Dolk et al., 2013) states that in the joint Simon task, each participant includes spatial codes of both their own and the other’s actions into their task set, because this helps distinguishing the actions of both persons sharing the task. If the relevant stimulus attribute is then presented on the other’s side, this activates the spatial response code linked with the other’s side and conflicts with one’s own response leading to the JSE. According to this account, the perceivable consequences of the other’s actions are used to shape the representation of one’s own individual task share according to the demands of the individual task share only (Dolk & Prinz, 2016).

A ubiquitous question is how behavior is optimized in joint action. In single tasks, different types of trial sequences have been used to investigate the optimization of behavior. For instance, conflict adaptation denotes a reduction of the compatibility effect following incompatible trials as compared with following compatible trials (e.g., Hommel et al., 2004; Notebaert & Soetens, 2001; Ridderinkhof, 2002; Stürmer et al., 2002) and has been interpreted as increased attentional selectivity following response conflicts (Botvinick et al., 2004).^1^ Similarly, the JSE was found to be reduced following incompatible trials both when the previous trial had been performed by oneself (go/go transitions) and even more so when it had been performed by the other (no-go/go transitions; Liepelt et al., 2013; Liepelt et al., 2011; Mendl et al., 2018; Yamaguchi et al., 2018). Because both, the action co-representation and the referential-coding accounts assume that response conflict is involved in the JSE, conflict adaptation following one’s own and following the other’s trials in the joint Simon task are in principle compatible with both accounts. Stronger conflict adaptation following the other’s trials can be explained by both accounts, if one assumes, for instance, that inhibition of one’s own response on the other’s trials increases response conflict leading to an amplification of adjustments on subsequent trials (cf. Yamaguchi et al., 2018). Alternatively, increased sequential effects following no-go trials can also be explained by so-called no-go tagging (e.g., Liepelt et al., 2011). Inhibition of one’s own response on no-go trials leads to a tag not to respond at the spatial location of the stimulus. If the stimulus on a subsequent go trial then occurs at the tagged location, this slows responses, because the inhibitory tag (i.e., the tendency not to respond) has to be overcome. Because the inhibitory tag is located at the current stimulus location on previous incompatible/current compatible and previous compatible/current incompatible no-go/go transitions, it slows responses on these trials. Although no-go-tagging explains stronger sequential effects without referring to response conflict on previous trials, both explanations are not mutually exclusive, and crucially, both assume that inhibition of one’s own response on the other’s trials is the reason for increased sequential effects on no-go/go transitions.

A straightforward test of the assumption that inhibition of one’s own response on the other’s trials produces the amplification of conflict adaptation on no-go/go transitions is to consider a situation where this inhibition obviously failed. This can be achieved easily in the joint Simon task, if one examines conflict adaptation following commission errors: If the other is in charge of responding on a given trial and responds correctly, but inhibition of one’s own response fails, a commission error by oneself on the other’s trial occurs. Hence, response inhibition cannot lead to the amplification of response conflict or to the establishment of an inhibitory tag. Therefore, one can predict that commission errors by oneself on the other’s trials should abolish the amplification of the conflict adaptation effect for no-go/go transitions.

The present study aimed at testing this hypothesis. We collected data from a joint Simon task and expected to obtain a typical JSE and postconflict adjustments that were amplified following the other’s correct responses compared with following one’s own correct responses as in previous studies (Klempova & Liepelt, 2016; Liepelt et al., 2013; Liepelt et al., 2011; Mendl et al., 2018; Yamaguchi et al., 2018). Crucially, we expected this amplification to be weakened or abolished following the other’s correct responses if one had additionally made a commission error. Because errors are infrequent in standard joint Simon tasks, which decreases the power to reveal posterror effects, we collapsed data across three experiments in which participants were given a speed instruction to obtain error rates higher than 15%. Experiment 1 used standard (neutral) joint Simon task instructions, Experiment 2 used cooperative, and Experiment 3 used competitive task instructions (e.g., Iani et al., 2011; Mendl et al., 2018). Because effects of instruction context on JSE and postconflict adjustments have previously been reported (e.g., Iani et al., 2011; Mendl et al., 2018), we report these effects although they were of less importance for the present purpose.

## Materials and methods

### Participants

Experiment 1 comprised 36 participants (25 female, 11 male, five left-handed) between 19 and 35 years of age (mean age = 23.5, *SE* = 0.672), Experiment 2 comprised 20 participants (18 female, two male, one left-handed) between 18 and 34 years of age (mean age = 22.2, *SE* = 0.847), and Experiment 3 comprised 20 participants (15 female, five male, three left-handed) between 18 and 28 years of age (mean age = 21.1, *SE* = 0.686). Some participant pairs had to be excluded from the analyses because either one or both participants did not commit any errors in at least one condition resulting in empty cells. As this was the case for one participant pair in Experiment 1, one participant pair in Experiment 2, and two participant pairs in Experiment 3, we obtained 34, 18, and 16 valid participants in Experiment 1, 2, and 3, respectively (68 valid participants in total). Note that this should result in sufficient power to detect the crucial three-way interaction (Current Compatibility × Previous Compatibility × Previous Actor) in posterror trials, where relatively low trial numbers are expected. Assuming an effect of 47 ms with a standard deviation of 28.66 ms (interaction term and standard deviation of interaction term calculated from Mendl et al., 2018), approximately 10 trials per condition are sufficient to reach acceptable power with little more than 60 participants (cf. Rouder & Haaf, 2018). All participants had normal or corrected-to-normal vision and were recruited from the student population at the university campus in Eichstätt. They received course credit or 8 Euro per hour for participation. Participants in Experiment 2 and 3 could additionally receive a performance-dependent reward. The study was conducted in accordance with the Declaration of Helsinki, and all participants gave informed consent.

### Stimuli and apparatus

Two PCs running Presentation software (Neurobehavioral Systems, Albany, CA) controlled stimulus presentation and response registration. Stimuli were presented on a 21-inch color monitor at a viewing distance of 80 cm. A small white cross-hair presented in the screen center served as fixation cross. The stimulus on each trial was either a green or a blue square, presented either on the left or on the right of the screen center. Stimuli subtended a visual angle of 0.74° × 0.74° (horizontal × vertical), and the horizontal distance between stimuli and the screen center was 4.44° at a viewing distance of about 80 cm. Color and location of the stimuli varied randomly from trial to trial, but each color and each location occurred with equal frequency across the experiment. Responses were given by pressing the left or the right ctrl. keys of a standard German QUERTZ computer keyboard.

### Task and procedure

Participants were invited in pairs to complete the task. They were seated side by side in front of the computer at equal distance from the screen center. Each participant was assigned one color (green or blue), to which they should respond. The assignment of colors to participants (left or right) was balanced across participants. The participants on the left (or on the right, respectively) were instructed to press the left “ctrl” key (or the right “ctrl” key) with the right hand, whenever the stimulus had the color they were each assigned to, and to ignore both the stimulus of the other color and the location of the stimulus (left, right). Each trial started with the presentation of the fixation cross for 250 ms. Then, the stimulus appeared for 150 ms, followed by a black screen. The first response triggered an interval of 1,000 ms during which the screen remained black before the next trial started. If any further responses occurred during this interval on a given trial (e.g., a commission error by the currently incorrect actor if the first response was a correct response by the currently correct actor, or a correct response by the currently correct actor if the first response was a commission error by the currently incorrect actor), they were also recorded. In this case, the ongoing interval of 1,000 ms was aborted, and a new interval of 1,000 ms was started before the next trial started.

Before the start of the experiment, participants first performed four blocks of a single go/no-go task with 24 trials each on separate computers. In this task, participants responded only to the color assigned to them in the later joint Simon task with the right hand but ignored the other color. Participants later seated on the left in the joint Simon task operated the left “ctrl” key with their right hand, and participants later seated on the right in the joint Simon task operated the right “ctrl” key with their right hand. Participants were instructed to respond as quickly as possible to the color of the stimulus without guessing. After each practice block, they were instructed to respond faster whenever their error rate in the preceding block was below 15%. This speed instruction was given to obtain a sufficient number of error trials for the analyses and was maintained for the whole experiment. After practicing the single Simon task, participants were seated in front of one computer side by side as described above and performed two practice blocks of the joint Simon task. Following practice, the experimental blocks started. Participants performed 20 blocks of 48 trials each preceded by three randomly drawn practice trials which were not considered in the data analyses. This resulted in a total of 960 trials per participant. The whole experiment lasted about 1 hr.

Task and procedure were identical for participants in Experiment 1, Experiment 2 and Experiment 3, except that in Experiment 2 and Experiment 3, participants were additionally given instructions as to the experimental context. In Experiment 2 (cooperative context), participant pairs were told that they would work together, and that each of them would be rewarded with additional 5 €, if their joint performance in terms of speed and accuracy of responses would be better than that of a normative sample. To evaluate joint performance, the joint inverse efficiency score [RT/(100 minus error rate) across the whole experiment] was calculated for each participant pair at the end of the experiment, and each participant was paid 5 €, if the participant pair’s joint inverse efficiency score was lower than the mean joint inverse efficiency score calculated across all participant pairs of Experiment 1. In Experiment 3 (competitive context), participant pairs were told that they would work together, and that only the participant with better performance in terms of speed and accuracy would be rewarded with additional 5 €. To evaluate each participant’s performance, the inverse efficiency score of each of the participants’ own responses was calculated at the end of the experiment, and only the participant with the lower inverse efficiency score was paid 5 €.

### Postexperiment survey

To assess whether the instructions of cooperation vs. competition in Experiment 2 and Experiment 3 successfully manipulated participants’ subjective experience, participants of Experiment 2 and Experiment 3 were asked to rate the experimental situation on a 7-point bipolar semantic differential scale on the following dimensions: easy–difficult (1 = *easy*, 7 = *difficult*), pleasant–unpleasant (1 = *pleasant*, 7 = *unpleasant*), positive–negative (1 = *positive*, 7 = *negative*), and cooperative–competitive (1 = *cooperative*, 7 = *competitive*).

### Data analyses

Data were analyzed using custom routines in MATLAB R2016a (The MathWorks, Natick, MA, USA). RT was defined as the time interval between the onset of the stimulus and the subsequent button press by the correct actor on a given trial. Trials containing commission errors by the incorrect actor were excluded for the analyses of RT. Errors were defined as responses by the incorrect actor on a given trial and used for the calculation of error rates. Conflict adaptation scores were calculated as [(current incompatible/previous compatible minus current compatible/previous compatible) minus (current incompatible/previous incompatible minus current compatible/previous incompatible)]. To control for outliers for the analyses of RT, two procedures were applied. First, trials with RT below 100 ms and above 1,500 ms were excluded (3.42%, *SE* = 0.255%) to remove fast guesses and very late responses, and second, trials on which RT was more than three standard deviations above or below the condition mean were excluded (0.652%, *SE* = 0.045%) to remove remaining outliers. Mean trial numbers per cell are given in Table 1. Because mean trial numbers following errors were rather low, we checked if trial numbers contributing to posterror trials differed between the previous self (12.42, *SE* = 0.674) and the previous other conditions (12.79, *SE* = 0.627). A paired-samples *t* test did not reveal a significant difference between these conditions, *t*(67) = 0.35, *p* = .725, *d*_*z*_ = 0.043. Error rates were arcsine-transformed before statistical testing (Winer et al., 1991). RT and transformed error rates were subjected to five-way mixed analyses of variance (ANOVAs) on the between-subjects variable group (Experiment 1, Experiment 2, Experiment 3), and the within-subjects variables current compatibility (current compatible, current incompatible), previous compatibility (previous compatible, previous incompatible), previous actor (previous self, previous other), and previous response (previous correct, previous error). We discuss only main effects not involved in any interaction and the highest significant interactions involving any particular variable. These interactions were further analyzed by decomposing them into separate lower level ANOVAs (for interactions involving more than two factors) and by planned comparisons using two-sided dependent samples *t* tests (for two-way interactions).

**Table 1:** Trial numbers

	Previous correct		Previous error	
	CC	CI	CC	CI
Previous Self				
PC	42.9 (1.22)	47.2 (1.41)	10.9 (0.763)	11.6 (0.733)
PI	44.6 (1.38)	40.5 (1.33)	14.1 (0.940)	13.0 (0.780)
Previous other				
PC	47.6 (1.26)	47.4 (1.47)	12.3 (0.854)	11.2 (0.678)
PI	44.5 (1.27)	45.6 (1.32)	13.5 (0.752)	14.1 (0.794)

CC = current compatible; CI = current incompatible; PC = previous compatible; PI = previous incompatible. Standard errors of the means are given in parenthesis.

Because unequal variances between groups could lead to a loss of power and/or inflation of test statistics for between-group comparisons, we checked for unequal variances between groups for all experimental conditions using the Levene test. No violations of the equality of variance assumption were detected. The lowest p-values were *p* = .117 for RT in the current compatible/previous incompatible/previous error/previous other condition (all other *p*s > .229) and *p* = .069 for arcsine-transformed error rates in the current compatible/previous incompatible/previous correct/previous other condition (all other *p*s > .167).

## Results

### Overall ANOVA for RT

**Fig. 1:**
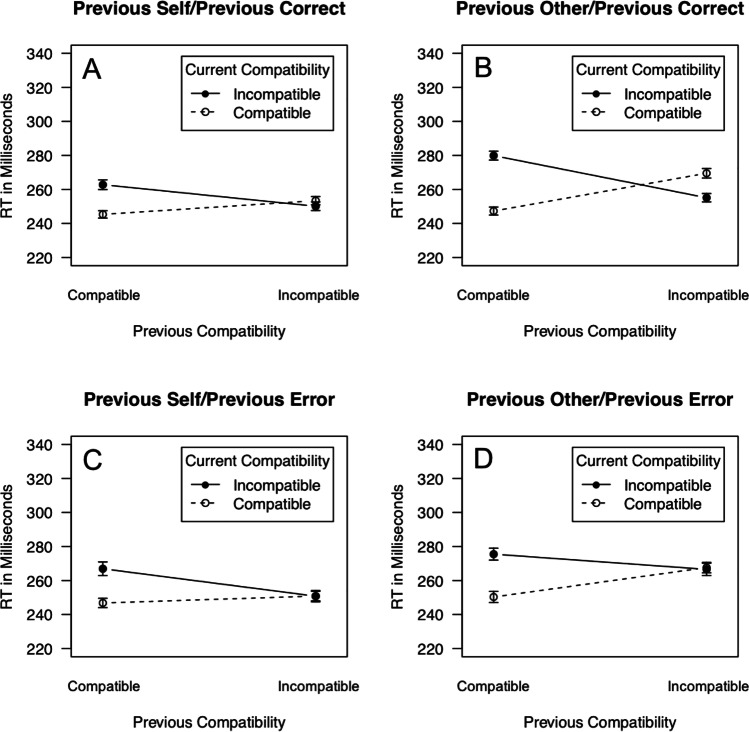
Response-time data: RT in milliseconds as a function of current compatibility (compatible, incompatible), previous compatibility (compatible, incompatible), previous actor (self, other), and previous response (correct, error). Error bars represent standard errors of the mean

The results of the overall ANOVA for RT are presented in Table 2. The main question of the present experiment was if the amplification of conflict adaptation following trials where the other was in charge of responding (previous other) was weakened for trials following errors (previous error). In line with this hypothesis, the overall ANOVA for RT revealed an interaction of current compatibility, previous compatibility, previous actor and previous response, *F*(1, 65) = 7.81, *p* = .007, ${\eta}_p^2$ = .107. The data are depicted in Fig. 1. Conflict adaptation effects (i.e., smaller or reversed compatibility effects following incompatible than following compatible trials) can be observed for previous self and for previous other. However, whereas for previous correct, conflict adaptation seems amplified for previous other as compared with previous self (Fig. 1b vs. Fig. 1a), such a pattern is not observable for previous error (Fig. 1d vs. Fig. 1c). To test this, we conducted separate three-way ANOVAs with the variables current compatibility, previous compatibility and previous actor for previous correct and for previous error. The overall ANOVA for RT had not revealed a main effect of the variable group, *F*(1, 65) = 1.36, *p* = .265, ${\eta}_p^2$ = .004, or interactions involving the variable group, all *F*s < 2.06, *p*s > .135, ${\eta}_p^2$s < .006.^2^ Therefore, the between-subjects variable group was dropped for the follow-up analyses. RT data for the experiments separately are listed in Table 3.

**Table 2:** ANOVA for RTs

Effect	*df*	MSE	*F*	*p*	${\eta}_p^2$
Group (G)	2, 65	4688	1.36	.265	.040
Current Compatibility (CC)	**1, 65**	**612**	**40.7**	**<.001**	**.385**
CC × G	2, 65	612	1.05	.357	.031
Previous Compatibility (PC)	1, 65	277.1	1.82	.182	.027
PC × G	2, 65	277.1	<1	.689	.011
Previous Actor (PA)	**1, 65**	**930**	**32.9**	**<.001**	**.336**
PA × G	2, 65	930	<1	.899	.003
Previous Response (PR)	1, 65	360.6	1.70	.197	.025
PR × G	2, 65	360.6	<1	.874	.004
CC × PC	**1, 65**	**228**	**243**	**<.001**	**.789**
CC × PC × G	2, 65	228	<1	.430	.026
CC × PA	1, 65	225.2	1.24	.269	.019
CC × PA × G	2, 65	225.2	1.03	.363	.031
PC × PA	**1, 65**	**256.2**	**8.38**	**.005**	**.114**
PC × PA × G	2, 65	256.2	<1	.800	.007
CC × PR	**1, 65**	**113**	**5.31**	**.024**	**.076**
CC × PR × G	2, 65	113	<1	.869	.004
PC × PR	1, 65	213.41	<1	.627	.004
PC × PR × G	2, 65	213.41	<1	.636	.014
PA × PR	1, 65	379	<1	.639	.003
PA × PR × G	2, 65	379	2.062	.135	.060
CC × PC × PA	**1, 65**	**394**	**11.2**	**.001**	**.148**
CC × PC × PA × G	2, 65	394	<1	.893	.003
CC × PC × PR	**1, 65**	**173.7**	**11.1**	**.001**	**.146**
CC × PC × PR × G	2, 65	173.7	1.81	.172	.053
CC × PA × PR	1, 65	193.1	<1	.993	<.001
CC × PA × PR × G	2, 65	193.1	1.66	.199	.048
PC × PA × PR	**1, 65**	**222**	**6.44**	**.014**	**.090**
PC × PA × PR × G	2, 65	222	1.03	.365	.031
CC × PC × PA × PR	**1, 65**	**222.9**	**7.81**	**.007**	**.107**
CC × PC × PA × PR × G	2, 65	222.9	<1	.720	.001

Significant effects at alpha = .05 are highlighted.

**Table 3:** Response times in milliseconds

	Previous correct			Previous error		
	CC	CI	Adaptation	CC	CI	Adaptation
Experiment 1						
Previous Self						
PC	248 (2.90)	265 (3.98)	20.7 (4.80)	252 (3.29)	271 (4.90)	17.9 (6.01)
PI	257 (2.66)	253 (3.45)		255 (3.77)	256 (4.24)	
Previous Other						
PC	253 (3.42)	283 (3.60)	45.4 (4.76)	255 (4.89)	274 (4.74)	20.9 (6.89)
PI	275 (4.02)	259 (3.54)		269 (3.59)	267 (5.15)	
Experiment 2						
Previous Self						
PC	241 (3.95)	265 (5.22)	26.2 (4.62)	244 (5.81)	262 (9.11)	18.3 (8.61)
PI	250 (4.83)	248 (5.16)		247 (5.36)	247 (6.37)	
Previous Other						
PC	242 (3.96)	279 (5.26)	50.8 (6.74)	246 (6.32)	282 (7.90)	30.8 (8.49)
PI	266 (5.07)	253 (4.69)		263 (5.69)	267 (8.10)	
Experiment 3						
Previous Self						
PC	245 (5.38)	255 (6.84)	12.9 (5.11)	238 (6.59)	264 (8.64)	28.4 (9.00)
PI	250 (6.94)	248 (5.21)		246 (6.61)	244 (8.68)	
Previous Other						
PC	242 (5.05)	273 (5.19)	45.5 (5.65)	244 (5.14)	269 (5.65)	32.5 (8.57)
PI	262 (5.65)	248 (4.43)		271 (9.23)	264 (5.09)	

CC = current compatible; CI = current incompatible; PC = previous compatible; PI = previous incompatible; Adaptation = (PC/CI minus PC/CC) minus (PI/CI minus PI/CC). Standard errors of the means are given in parenthesis.

#### Follow-up analyses for RT following correct responses

The ANOVA for trials following correct responses revealed an interaction of current compatibility, previous compatibility and previous actor, *F*(1, 67) = 32.4, *p* < .001, ${\eta}_p^2$ = .326. Two separate two-way ANOVAs for previous self/previous correct and previous other/previous correct with the variables current compatibility and previous compatibility revealed interactions of current compatibility and previous compatibility both for previous self/previous correct, *F*(1, 67) = 48.6, *p* < .001, ${\eta}_p^2$ = .420, and for previous other/previous correct, *F*(1, 67) = 206, *p* < .001, ${\eta}_p^2$ = .754 (previous compatibility *p* = .158, ${\eta}_p^2$ = .029). The fact that both interactions were significant denotes that conflict adaptation did occur in both cases. Thus, the interaction of current compatibility, previous compatibility and previous actor can be explained by stronger conflict adaptation scores for previous other/previous correct (47.0 ms, *SE* = 3.28 ms, cf. Fig. 1b) than for previous self/previous correct (20.7 ms, *SE* = 2.97 ms, cf. Figure 1a).

#### Follow-up analyses for RT following errors

The ANOVA for previous error revealed interactions of current compatibility and previous compatibility, *F*(1, 67) = 72.4, *p* < .001, ${\eta}_p^2$ = .519, denoting conflict adaptation, and of previous compatibility and previous actor, *F*(1, 67) = 8.61, *p* < .005, ${\eta}_p^2$ = .114. RT was faster for previous self/previous error than for previous other/previous error both when the previous trial was compatible (previous self/previous error: 257 ms, *SE* = 2.99 ms; previous other error: 263 ms, *SE* = 3.01 ms), *t*(67) = 2.30, *p* = .025, *d*_*z*_ = 0.279, and when the previous trial was incompatible (previous self/previous error: 251 ms, *SE* = 2.72 ms; previous other/previous error: 267 ms, *SE* = 2.83 ms), *t*(67) = 4.98, *p* < .001, *d*_*z*_ = 0.604. Thus, the interaction of previous compatibility and previous actor indicated that the shortening of RT for previous self/previous error was more pronounced following incompatible than following compatible trials. Most importantly, however, the ANOVA for previous error trials did not reveal an interaction of current compatibility, previous compatibility and previous actor, *F*(1, 67) = .733, *p* = .395, ${\eta}_p^2$ = .011. Thus, for previous error, conflict adaptation did occur, but was not affected by previous actor (previous other/previous error: 26.2 ms, *SE* = 4.59 ms, cf. Fig. 1d; previous self/previous error: 20.2 ms, *SE* = 4.31 ms, cf. Fig. 1c).

#### Control analyses for RT

It has been reported that conflict adaptation tends to decay for very long ITIs (e.g., Duthoo et al., 2014). For instance, using a Stroop task, Egner et al. (2010) showed linearly decreasing conflict adaptation effects when ITIs increased in bins from 500 ms to 7,000 ms with conflict adaptation effects being nonsignificant for ITIs over 4,000 ms. This could be relevant in the present context because our ITIs varied with RT due to the fixed response-stimulus interval of 1,000 ms inserted after the last response. The main result of the present study was that conflict adaptation was weaker for previous self/previous correct than for previous other/previous correct, whereas conflict adaptation did not differ between previous self/previous error and previous other/previous error. This pattern could arise due to decaying conflict adaptation for longer preceding ITIs in one of two ways. First, if preceding ITIs were longer for previous self/previous correct than for previous other/previous correct this could weaken conflict adaptation for previous self/previous correct. Second, if preceding ITIs were longer for previous other/previous error than for previous self/previous error conflict adaptation could be weakened for previous other/previous error. To investigate if this was the case, we analyzed the preceding ITIs for these conditions. We found no significant difference between previous self/previous correct (2,134 ms, *SE* = 76.9 ms) and previous other/previous correct (2,134 ms, *SE* = 76.7 ms), *F*(1, 65) = 0.013, *p* = .910, ${\eta}_p^2$ < .001. This shows that longer previous ITIs for previous self/previous correct than for previous other/previous correct cannot be the reason for weaker conflict adaptation for previous self/previous correct. Similarly, we found no significant difference between previous self/previous error and previous other/previous error, *F*(1, 65) = 2.616, *p* = .111, ${\eta}_p^2$ = .039. If anything, previous ITIs were longer for previous self/previous error (2,226 ms, *SE* = 82.1 ms) than for previous other/previous error (2,188 ms, SE = 80.3 ms) showing that conflict adaptation cannot have been weakened due to longer previous ITIs for Previous other/previous error as compared with previous self/previous error. Thus, we feel confident that our results do not reflect decaying conflict adaptation due to varying previous ITIs between conditions.

### Overall ANOVA on error rates

**Fig. 2:**
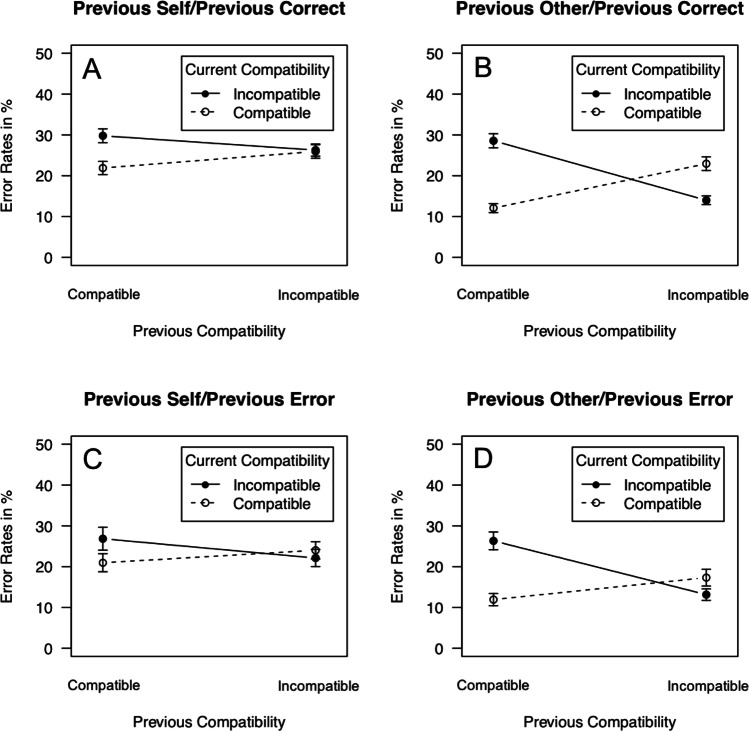
Error rates: Error Rates in % as a function of current compatibility (compatible, incompatible), previous compatibility (compatible, incompatible), previous actor (self, other), and previous response (correct, error). Error bars represent standard errors of the mean.

Error rates are shown in Fig. 2, and the results of the overall ANOVA for error rates are presented in Table 4. The ANOVA revealed an interaction of current compatibility, previous compatibility and previous actor, *F*(1, 65) = 27.7, *p* = .001, ${\eta}_p^2$ = .299, denoting that previous actor modulated conflict adaptation: Conflict adaptation was found both for previous self [Current Compatibility × Previous Compatibility: *F*(1, 65) = 15.2, *p* < .001, ${\eta}_p^2$ = .189] and previous other [Current Compatibility × Previous Compatibility: *F*(1,65) = 117, *p* < .001, ${\eta}_p^2$ = .643], but was stronger for previous other (22.0%, *SE* = 1.94%, see Fig. 2 and 2d) than for previous self (7.68%, *SE* = 1.78%, see Fig. 2a, 2c). Thus, error rates showed conflict adaptation, which was enhanced for previous other. However, unlike for RT, the ANOVA for error rates did not reveal an interaction of Current Compatibility, Previous Compatibility, previous actor and previous response, *F*(1, 65) = 2.07, *p* = .155, ${\eta}_p^2$ = .031. Thus, in the error rates, the amplification of conflict adaptation for previous other/previous error was not significantly dampened compared with previous self/previous error. Finally, the error rate was higher for previous correct (22.7%, *SE* = 1.06%) than for previous error (20.3%, *SE* = 1.28%), *F*(1, 65) = 13.8, *p* < .001, ${\eta}_p^2$ = .175. Participants responded more accurately both for previous self/previous error than for previous self/previous correct (previous self/previous correct minus previous self/previous error = 2.48%, *SE* = 1.17%), *t*(67) = 3.45, *p* < .001, *d*_*z*_ = 0.418, and for previous other/previous error than for previous other/previous correct (previous other/previous correct minus previous other/previous error = 2.21%, se = 1.30%), *t*(67) = 2.80, *p* < .01, *d*_*z*_ = 0.339. Error rate data for the experiments separately are listed in Table 5.

**Table 4:** ANOVA for error rates

Effect	*df*	MSE	*F*	*p*	${\eta}_p^2$
Group (G)	2, 65	0.874	0.528	.593	.016
Current Compatibility (CC)	**1, 65**	**0.162**	**15.4**	**<.001**	**.191**
CC × G	2, 65	0.162	<1	.810	.006
Previous Compatibility (PC)	1, 65	0.117	1.86	.178	.028
PC × G	2, 65	0.117	<1	.419	.026
Previous Actor (PA)	**1, 65**	**0.289**	**28.7**	**<.001**	**.306**
PA × G	2, 65	0.289	<1	.891	.004
Previous Response (PR)	**1, 65**	**0.194**	**13.8**	**<.001**	**.175**
PR × G	2, 65	0.194	<1	.812	.006
CC × PC	**1, 65**	**0.072**	**135**	**<.001**	**.674**
CC × PC × G	2, 65	0.072	<1	.430	.004
CC × PA	1, 65	0.088	2.36	.130	.035
CC × PA × G	2, 65	0.088	1.205	.306	.036
PC × PA	**1, 65**	**0.096**	**4.54**	**.037**	**.065**
PC × PA × G	2, 65	0.096	1.60	.210	.047
CC × PR	1, 65	0.073	<1	.611	.004
CC × PR × G	2, 65	0.073	<1	.841	.005
PC × PR	1, 65	0.105	<1	.512	.007
PC × PR × G	2, 65	0.105	<1	.433	.025
PA × PR	1, 65	0.089	<1	.766	.001
PA × PR × G	2, 65	0.089	1.32	.274	.039
CC × PC × PA	**1, 65**	**0.097**	**27.7**	**.001**	**.299**
CC × PC × PA × G	2, 65	0.097	<1	.675	.012
CC × PC × PR	1, 65	0.088	1.23	.272	.019
CC × PC × PR × G	2, 65	0.088	<1	.750	.009
CC × PA × PR	1, 65	0.068	2.34	.131	.035
CC × PA × PR × G	2, 65	0.068	<1	.378	.029
PC × PA × PR	1, 65	0.084	<1	.014	.009
PC × PA × PR × G	2, 65	0.084	1.14	.365	.043
CC × PC × PA × PR	1, 65	0.063	2.074	.156	.031
CC × PC × PA × PR × G	2, 65	0.064	1.65	.200	.048

Significant effects at alpha = .05 are highlighted.

**Table 5:** Error rates in %

	Previous correct			Previous error		
	CC	CI	Adaptation	CC	CI	Adaptation
Experiment 1						
Previous Self						
PC	22.0 (2.41)	28.3 (2.16)	7.39 (3.13)	18.8 (3.02)	23.6 (3.65)	8.95 (3.90)
PI	26.0 (2.56)	24.9 (2.09)		24.1 (3.56)	20.0 (2.77)	
Previous Other						
PC	10.8 (1.33)	27.4 (2.35)	24.8 (3.19)	10.9 (2.23)	24.1 (2.90)	18.7 (3.52)
PI	21.5 (2.46)	13.3 (1.35)		19.0 (3.21)	13.4 (2.03)	
Experiment 2						
Previous Self						
PC	21.2 (2.69)	30.4 (3.14)	4.75 (3.44)	23.6 (4.70)	34.3 (5.79)	10.8 (5.40)
PI	22.8 (2.18)	27.3 (2.90)		23.1 (2.84)	23.1 (3.98)	
Previous Other						
PC	12.8 (2.17)	28.8 (2.83)	25.9 (3.53)	13.1 (2.94)	24.6 (3.98)	14.4 (4.93)
PI	23.8 (3.47)	13.9 (2.05)		16.1 (3.69)	13.1 (2.64)	
Experiment 3						
Previous Self						
PC	22.5 (3.85)	32.4 (4.66)	12.0 (4.72)	22.6 (4.20)	24.1 (6.10)	0.699 (7.98)
PI	30.2 (4.00)	28.1 (3.29)		25.0 (4.05)	25.9 (5.36)	
Previous Other						
PC	14.1 (2.87)	31.1 (4.75)	26.4 (5.15)	12.7 (2.55)	34.2 (5.22)	24.05 (7.61)
PI	25.3 (2.68)	15.9 (2.85)		15.0 (3.53)	12.5 (3.12)	

CC = Current Compatible; CI = Current Incompatible; PC = Previous Compatible; PI = Previous Incompatible; Adaptation = (PC/CI minus PC/CC) minus (PI/CI minus PI/CC). Standard Errors of the Means Are Given in Parenthesis

## Discussion

The present study investigated adaptive adjustments of behavior in joint action. Previous studies showed that the JSE in the joint Simon task is reduced following incompatible trials, where the actors themselves have responded and even more so following incompatible trials where the other persons have responded (Liepelt et al., 2013; Liepelt et al., 2011; Mendl et al., 2018; Yamaguchi et al., 2018). This is surprising, because there is no obvious reason to adjust one’s behavior more on trials following action observation than on trials following action execution. However, two not mutually exclusive ideas have been advocated to explain this effect. One was that inhibition of one’s own response on *other* trials increases response conflict leading to enhanced conflict adaptation on no-go/go transitions (cf. Yamaguchi et al., 2018). A second was that inhibition of one’s own response on *other* trials leads to a spatial inhibitory tag at the location of the stimulus, which is then reactivated on the upcoming trial slowing responses (e.g., Liepelt et al., 2011). For instance, if the other seated on the left responds correctly to a stimulus on the left (preceding trial compatible no-go), one inhibits one’s own response to stimuli on the left, which leads to no-go-tagging of left stimuli. This inhibitory tag of the left side needs to be overcome on upcoming incompatible go trials (own side right, stimulus on the left), but not on upcoming compatible go trials (own side right, stimulus on the right). This slows RT on compatible/incompatible no-go/go transitions. Conversely, if the other on the left responds correctly to a stimulus on the right (preceding trial incompatible no-go), one inhibits one’s own response to stimuli on the right and the resulting right side inhibitory tag needs to be overcome on upcoming compatible go trials (own side right, stimulus on the right), but not on upcoming incompatible trials (own side right, stimulus on the left). This slows RT on incompatible/compatible no-go/go transitions (even leading to a reversal of the compatibility effect on these trials, Fig. 1b).

Thus, both amplification of response conflict by inhibition of one’s own response and no-go-tagging on no-go/go transitions assume that inhibition of one’s own response is the reason why conflict adaptation is enhanced following *other* trials. We tested this idea by asking what happens if one makes commission errors on no-go/go transitions (i.e., the inhibition of one’s own response on these trials fails). We observed amplified conflict adaptation for no-go/go transitions where the other had responded correctly and oneself had correctly withheld the response. Crucially, such an amplification of conflict adaptation was abolished when commission errors by oneself were present on no-go/go transitions. This result supports our hypothesis that if inhibition of one’s own response fails on no-go trials, response conflict is not enhanced/no inhibitory tag is established and responses on upcoming go trials remain unaffected. Commission errors by the other on previous trials did not affect conflict adaptation on go/go transitions. This is also in line with our hypothesis. On go/go transitions, no inhibition of one’s own responses is necessary and therefore, response conflict is not enhanced/no inhibitory tag is established on these trials. Interestingly, conflict adaptation in the error rates was amplified on no-go/go transitions irrespective of the presence of errors on no-go trials. This may indicate that amplification of response conflict/no-go-tagging in fact occur to some extent even if inhibition of one’s own response fails.

Response inhibition as an important factor in joint action also fits with the general idea of referential coding: If inhibition of one’s own response on preceding *other* trials is successful, a link between the spatial location of the preceding stimulus and representations such as “other,” “spatial location of the other’s response,” “not responding oneself,” and so forth, is established. If the stimulus location repeats on upcoming *self* trials, this leads to the retrieval of these conflicting representations, which slows responding (cf. Yamaguchi et al., 2018, p.392). By contrast, if inhibition fails, the spatial location of the preceding stimulus is linked to representations like “self,” “spatial location of one’s own response,” or “responding oneself.” This eliminates or at least weakens the link with the representations related to the other and their detrimental effects on performance.

A crucial role of response inhibition in joint action is in accord with previous reports of enhanced frontocentral no-go P3 amplitudes to observed actions in joint contexts (Sebanz et al., 2006b; Tsai et al., 2006). In single tasks, the frontocentral no-go P3 is thought to reflect motor inhibition on no-go trials (e.g., Pfefferbaum et al., 1985; Smith et al., 2008). Similarly, this component was interpreted as the activation and subsequent inhibition of one’s own action on trials by the other in joint action (Sebanz et al., 2006a).

We did not replicate the effect of cooperative and competitive contexts on conflict or conflict adaptation that was seen in previous studies. In these studies, smaller compatibility effects (Iani et al., 2011) and stronger conflict adaptation on go/go transitions (Mendl et al., 2018) were observed under competitive vs cooperative instructions, presumably due to lower attention paid to the other in competitive joint action contexts. As we employed much higher time pressure as these previous studies—error rates were 1.3% or lower in previous studies as opposed to 21.5% in the present study—this might suggest that high time pressure limits the effectiveness of such context effects in joint action. In any case, we found typical JSEs and postconflict adjustments in RT and error rates despite the high time pressure in our experiments. This indicates that, first, these effects are robust in RT also with high time pressure, and second, they are observable also in the error rates. This is a novel result because previous studies did not analyze the JSE or postconflict adjustments in error rates because these were rather low (e.g., 1.3% to 2.2 % in the studies by Liepelt et al., 2013; Liepelt et al., 2011; Mendl et al., 2018; Yamaguchi et al., 2018).

One further finding of the present study was that error rates were lower following errors than following correct responses irrespective of the actor on the previous trial. This shows that both following own errors on no-go/go transitions and following errors by the other on go/go transitions, one adaptively adjusts one’s own behavior and responds more accurately. Similar adaptive posterror adjustments were also reported in previous studies on joint action (De Bruijn et al., 2012; Núñez Castellar et al., 2011; Schuch & Tipper, 2007), as well as in single tasks (Danielmeier et al., 2011; Laming, 1968; Maier et al., 2015; Maier & Steinhauser, 2016; Maier et al., 2011; Marco-Pallarés et al., 2008). This points to a generic system for error monitoring and posterror adjustments of behavior that supports the optimization of behavior in solo action, as well as in joint action.

A limitation of the present study could be that commission errors could have occurred due to reasons other than failures of response inhibition, such as failures of attending to the stimulus, misinterpretations of the stimulus, or failures of task engagement. However, in our study, participants responded more accurately both following commission errors by the other on preceding Self trials and following own commission errors on preceding Other trials (see also, Schuch & Tipper, 2007), suggesting that both errors were reliably detected. Moreover, in a joint go/no-go paradigm comparable to ours, the error-related negativity - a neural correlate of error monitoring that requires a representation of the correct response (see, e.g., Di Gregorio et al., 2018) - was found not only on Self trials where the other person committed an error, but also on *other* trials, where oneself committed an error (de Bruijn et al., 2011). Both observations suggest that the no-go stimulus must have been correctly identified on most of the errors on *other* trials. This makes it unlikely that a substantial portion of commission errors occurred due to failures of attending to the stimulus, misinterpretations of the stimulus, or failures of task engagement. Therefore, although we cannot exclude that some errors on preceding *other* trials were due to other reasons, we believe that failures of response inhibition, i.e., actual commission errors where the stimulus was correctly identified, were the dominant error source in our data.

In sum, we showed that inhibition of one’s own responses on the other’s trials reflects an integral component of action control in joint action. Our data are in line with the view that successful inhibition of own responses contributes to shaping representations of one’s own task share. This view has previously been called *task shaping in joint action* combining individual and social elements (Prinz, 2015; Wenke et al., 2011).

## Footnotes

1 Note that it was also argued that sequential modulations of the Simon effect may be due to trial-to-trial repetitions and alternations of task features, which are confounded with the compatibility sequence (Hommel et al., 2004). This account was also advocated for sequential modulations of the JSE (Liepelt et al., 2011).

2 Also, when only RT data from Experiment 2 and Experiment 3 were considered, the variable *group* did not yield a significant main effect, *F*(1, 32) = 0.152, *p* = .699, ${\eta}_p^2$ = .005, or significant interactions involving the variable Group, all *F*s < 2.88, *p*s > .099, ${\eta}_p^2$s < .0823. This shows that competitive vs. cooperative context did not affect RT. However, questionnaire results indicated that contexts were at least subjectively effective. Mean ratings did not differ between groups for the dimensions easy–difficult, pleasant–unpleasant, and positive–negative. By contrast, participants in the competitive group rated the experimental situation as more competitive (3.17 points, *SE* = 0.405 points) than did participants in the cooperative group (2.15 points, *SE* = 0.196 points), *t*(30) = 2.53, *p* = .017, *d* = 0.925.
